# Papillome inversé: étude rétrospective à propos de 22 cas

**DOI:** 10.11604/pamj.2014.17.208.3936

**Published:** 2014-03-16

**Authors:** Mehdi Chihani, Karim Nadour, Mohamed Touati, Youssef Darouassi, Haddou Ammar, Brahim Bouaity

**Affiliations:** 1 Service d'Oto-Rhino-Laryngologie et Chirurgie Cervico-Faciale, Hôpital Militaire Avicenne, Marrakech, Maroc

**Keywords:** Papillome inversé, tumeur naso-sinusienne, endoscopie, imagerie, chirurgie endoscopique, Inverted papilloma, naso-sinus tumor, endoscopy, imagery, endoscopic surgery

## Abstract

Le papillome inversé est une tumeur bénigne naso-sinusienne rare, marquée par une forte agressivité locale, un taux élevé de récidive après chirurgie et un risque imprévisible d'association à un carcinome épidermoïde. Il s'agit d'une étude rétrospective de 22 cas de papillome inversé, colligés entre janvier 2000 et décembre 2012 au service d'oto-rhino-laryngologie et chirurgie cervico-faciale de l'hôpital militaire Avicenne de Marrakech. L'objectif de ce travail est d’étudier le profil épidémiologique, clinique, endoscopique, radiologique, thérapeutique et évolutif du papillome inversé. Le sex-ratio a été de 3,7 en faveur du sexe masculin avec une moyenne d’âge de 44 ans et un pic de fréquence entre la quatrième et la cinquième décade. Les symptômes cliniques ont été dominés par l'obstruction nasale. Le bilan radiologique faisant appel au couple TDM et IRM naso-sinusiennes constitue un moyen essentiel pour le diagnostic positif et dans choix de la technique opératoire. La voie vestibulaire sous labiale de Rouge Denker a été utilisée chez 4 patients, 12 patients ont bénéficié d'une chirurgie endoscopique endonasale et 6 patients d'une combinaison des deux voies précédentes. Cinq patients ont eu une récidive du papillome inversé, après un délai moyen de 26 mois.

## Introduction

Le papillome inversé est une tumeur épithéliale bénigne d’évolution lente. Elle est de cause encore incertaine. Elle se singularise des autres tumeurs bénignes des cavités nasales par trois caractères importants: un haut potentiel d'agressivité locale et régionale, un fort degré de récidive après traitement et un risque imprévisible d'association à un carcinome épidermoïde. L'objectif de cette étude est d’évaluer les caractéristiques épidémiologiques et cliniques, et de préciser les différentes modalités thérapeutiques et évolutives de la maladie.

## Méthodes

Il s'agit d'une étude rétrospective étalée sur 13 ans, allant du janvier 2000 au décembre 2012, et concerne 22 cas de papillome inversé colligés au sein du service d'ORL et de chirurgie cervico-faciale de l'hôpital militaire Avicenne de Marrakech. Le diagnostic a été posé sur des arguments cliniques, radiologiques et anatomo-pathologiques.

## Résultats

La série comporte 22 patients, 6 femmes et 16 hommes. Il y'avait une nette prédominance masculine avec un sex-ratio homme/femme de 3,7. Les patients étaient âgés de 24 à 62 ans, avec une moyenne d’âge de 44 ans et un pic de fréquence entre la quatrième et la cinquième décade. Trois patients avaient comme antécédents une rhinite allergique. Le signe fonctionnel le plus fréquent était l'obstruction nasale, trouvée chez 18 patients, soit 81,8% (Tableau 1). L'endoscopie nasale a montré une atteinte unilatérale chez tous les malades. L'aspect de la tumeur était grisâtre polylobé chez 13 cas (59,1%), saignant au contact dans un cas (4,5%), translucide chez 3 cas (13,6%), alors que le caractère évocateur en grappe de raisin n'a été retrouvé que chez 5 patients (22,7%). L'origine de la tumeur était difficile à préciser, mais tous les patients avaient une composante intra-nasale de la tumeur, des sécrétions purulentes dans 7 cas (31,8%), une déviation septale dans 6 cas (27,2%) et une hypertrophie turbinale bilatérale chez 2 patients (9,1%).

**Tableau 1:** Les différents signes fonctionnels retrouvés dans la série

Signes fonctionnels	Nombre de cas	Fréquence (%)
Obstruction nasale	18	81,8
Rhinorrhée	8	36,3
Epistaxis	5	22,7
Dysosmie	4	18,1
Algie faciale	3	13,6
Céphalées	2	9,1

**Figure 1:**
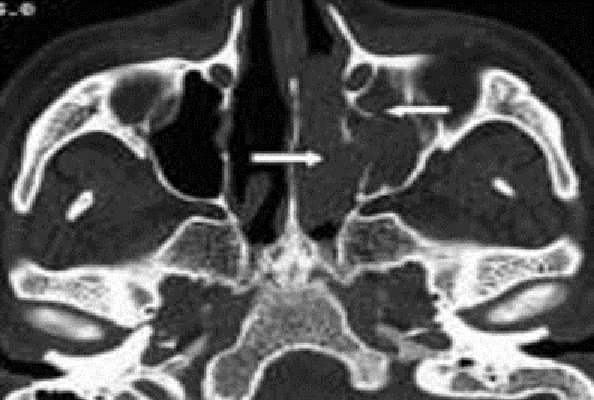
TDM naso-sinusienne en coupe axiale en fenêtre osseuse montrant un processus tissulaire isodense de la fosse nasale gauche étendu au sinus maxillaire homolatéral avec destruction de la paroi inter-sinuso-nasale

**Figure 2:**
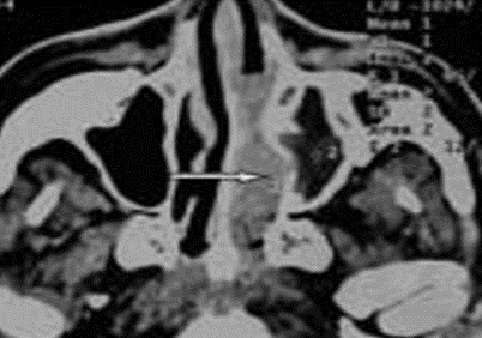
TDM naso-sinusienne en coupe axiale montrant un processus tissulaire hypodense de la fosse nasale gauche avec refoulement de la paroi inter-sinuso-nasale

**Figure 3:**
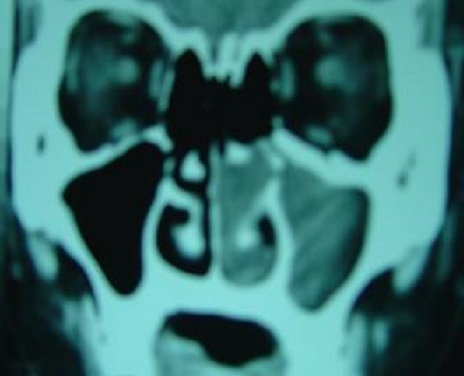
TDM naso-sinusienne en coupe coronale montrant un processus tissulaire comblant la fosse nasale et le sinus maxillaire du côté gauche

Une tomodensitométrie (TDM) naso-sinusiennes a été réalisée chez tous les patients. L'aspect radiologique retrouvé avant l'injection de produit de contraste variait entre des images tissulaires isodenses dans 15 cas et hypodenses dans 7 cas. Après injection de produit de contraste, ce produit se rehaussait de façon hétérogène dans 17 cas et prenait faiblement le contraste dans 5 cas. Les lésions ont été unilatérales dans tous les cas: elles intéressait le côté gauche dans 12 cas (54,5%), contre 10 cas pour le côté droit (45,5%). Le sinus maxillaire était atteint dans 15 cas, le sinus ethmoïdal dans 5 cas et le cavum a été envahi dans 3 cas. Une ostéolyse était présente dans 6 cas (Figure 1, Figure 2, Figure 3).

Pour l'imagerie par résonance magnétique (IRM), personne de nos malades n'en avait bénéficié.

Un bilan biologique a été réalisé chez tous les patients comportant un hémogramme, un groupage sanguin, un bilan d'hémostase et un ionogramme sanguin complet. Le bilan allergologique, notamment les tests cutanés et le dosage des Ig E spécifiques, n'a pas été effectué chez nos patients.

La biopsie sous guidage endoscopique a été réalisée chez tous les patients. Elle a confirmé le diagnostic de papillome inversé dans les deux tiers des cas (15 cas). Dans les cas restants elle a été en faveur de polypes inflammatoires sans aucune spécificité.

Le choix de la voie d'abord a été dicté par le bilan d'extension de la tumeur évalué par l'examen endoscopique et le bilan radiologique: 4 patients ont subi un traitement par voie vestibulaire sous labiale de Rouge Denker, 12 patients ont bénéficié d'une chirurgie endoscopique endonasale et 6 patients ont été opérés par combinaison des deux voies précédentes. Tous nos patients ont bénéficié d'une exérèse totale de la tumeur et de la muqueuse de la paroi externe de la fosse nasale, associée à une résection de la muqueuse du sinus maxillaire homolatérale chez 15 patients et à une ethmoïdectomie dans 5 cas. Tous les patients ont nécessité un méchage nasal antérieur bilatéral.

L'examen anatomopathologique des pièces d'exérèse a confirmé le diagnostic de papillome inversé dans tous les cas. Aucun signe de malignité histologique n'a été noté. Le déméchage a été réalisé au 2ème-3ème jour du post-opératoire, suivi par des lavages pluriquotidiens des fosses nasales, à l'aide du sérum physiologique, entrepris et maintenus pendant un mois.

Les suites post-opératoires étaient marquées par la survenue d’épistaxis de moyenne abondance dans 4 cas qui ont nécessité un reméchage pendant 48h, une infection de la cavité nasale a été notée dans 3 cas qui ont été traités par une antibiothérapie à base d'amoxicilline protégée à la dose de 3 g/j.

Les contrôles sont effectués à 1 mois, 3 mois et 6 mois, puis annuellement tout au long de la période d’étude. La surveillance post-opératoire s’étale sur une période variant entre 6 mois et 11 ans, au terme de laquelle nous avons noté une bonne évolution chez 14 malades, 5 cas de récidive ont été notés avec un délai moyen de 26 mois. La reprise de ces 5 cas a consisté en une exérèse tumorale par voie endoscopique exclusive dans 3 cas et couplée à une voie vestibulaire dans les deux autres cas. Les contrôles endoscopiques post-opératoires ultérieurs n'ont objectivé aucune récidive avec un recul de 4 ans.

## Discussion

Actuellement, la fréquence des papillomes inversés rapportée dans la littérature correspond à 0.5 à 4% des tumeurs primitives des cavités naso-sinusiennes [1, 2]. Le papillome se rencontre préférentiellement chez l'homme avec un sex-ratio de 3 à 4 [3]. L’âge de découverte se situe entre 5 et 70 ans avec la possibilité de cas diagnostiqués chez de jeunes adultes et même de façon exceptionnelle chez l'enfant [4].

La symptomatologie est commune avec les autres tumeurs naso-sinusiennes, le maître symptôme étant l'obstruction nasale unilatérale. D'autres manifestations cliniques variées, en rapport avec la topographie et l'extension tumorale, peuvent exister: rhinorrhée, épistaxis, douleurs faciales, céphalées frontales, anosmie, diplopie, otalgie [5], voire même des déformations faciales en rapport avec l'agressivité locale de cette tumeur. Aucun symptôme n'est spécifique, tout en insistant sur l'unilatéralité de la symptomatologie qui devra attirer l'attention.

L'examen endoscopique montre une tumeur prenant un aspect exophytique et polyploïde, pouvant présenter une extension vers le nasopharynx à travers les choanes, de couleur grise à rose. La tumeur peut être friable ou hémorragique au décours d'une biopsie. Elle peut être masquée par un polype sentinelle. Il n'existe pas de coté de prédilection pour le papillome inversé qui est en règle générale unilatéral. Sa localisation la plus fréquente est la paroi latérale des fosses nasales (80%), plus particulièrement la région du méat et du cornet moyen, envahissant le complexe ostio-méatal. Occasionnellement, le septum peut être atteint de même que le cornet et le méat inférieur. D'autres sites anatomiques peuvent être touchés comme le sinus maxillaire ou l'ethmoïde. Plus rarement, la tumeur peut avoir pour point de départ le sinus frontal et de façon plus exceptionnelle le sinus sphénoïde. Les papillomes inversés bilatéraux sont rares et représentent 4 à 5% des patients [6, 7].

Les imageries de référence sont la TDM et l'IRM. En TDM, le papillome inversé se présente comme une masse polyploïde, spontanément isodense aux tissus mous, centrée sur le méat moyen, s’étendant au carrefour ostio-méatique et au sinus maxillaire homolatéral. Cette masse possède des contours lobulés, et présente un rehaussement hétérogène après injection, ce qui le différencie des sécrétions rétentionnelles [8]. Le papillome inversé peut être le siège de petites calcifications, initialement décrites comme faisant partie de la matrice tumorale. Il est actuellement admis qu'il s'agit en fait de débris osseux piégés au sein de la masse. Même si en théorie, la prise de contraste tumorale est hétérogène alors qu'elle est périphérique en cas d'atteinte inflammatoire, ces deux composantes sont fréquemment intriquées, et il est difficile de faire la part des choses sur un examen tomodensitométrique. C'est pour cette raison que la délimitation précise de la composante tumorale est malaisée et que la taille tumorale est souvent surestimée au scanner [8]. En résumé, le scanner de première intention permet de préciser le siège du papillome inversé et son caractère lobulé en surface, d’évaluer son extension ainsi que l'atteinte osseuse associée. L'IRM est l'outil indispensable au diagnostic et à l’évaluation pré-thérapeutique précise de toute tumeur naso-sinusienne. Elle permet une étude plus fine de l'extension tumorale par rapport aux structures adjacentes et différencie la tumeur d'une image rétentionnelle. La séquence T2 distingue le papillome inversé (de signal intermédiaire) du tissu polypeux adjacent ou d'une rétention liquidienne (hypersignal). En séquence T1 injecté, le papillome inversé se rehausse de manière homogène. Ceci semble particulièrement important dans les régions frontale et maxillaire car cela conditionne la voie d'abord choisie [9].

Le traitement des papillomes inversés est chirurgical. Leur haut potentiel d'agressivité locale, la crainte d'une récidive et la possibilité d'une association à une tumeur maligne imposent une prise en charge radicale. À l'heure actuelle, aucun consensus n’étant établi quant à la technique chirurgicale à adopter, la ou les voies d'abord doivent être choisies en vue d'une exérèse carcinologique de la tumeur, qu'elle soit bénigne dans sa totalité ou présentant déjà des signes de malignité. Actuellement, on peut considérer que les voies d'abord externes trans-faciales et endoscopiques sont complémentaires. La voie endoscopique permet de traiter les papillomes inversés qui se développent au niveau de l'ethmoïde et du sphénoïde et éventuellement les extensions très limitées à la partie inférieure du sinus frontal [4, 10]. L'extension au sinus frontal avec syndrome de masse tumoral nécessite une bonne exposition pour une exérèse satisfaisante des lésions, d'où le choix entre voie coronale et voie sourcilière, en fonction de l'implantation de la racine des cheveux et de l'avis du patient. La voie para-latéro-nasale n'apporte pas des avantages décisifs sur une exérèse endonasale pour la partie ethmoïdale de la tumeur. Lorsqu'il y a une atteinte maxillaire, si elle n'est pas trop externe et/ou antérieure, la voie endonasale avec méatotomie moyenne associée éventuellement à une maxillectomie partielle endoscopique, permet un bon contrôle tumoral. En revanche, l'abord endoscopique de la fosse canine peut toujours être nécessaire pour l'exérèse complète des lésions intra-maxillaires, car seule cette voie permet une exploration complète des trois récessus lacrymal, orbitaire et zygomatique du sinus maxillaire et une exploration correcte de sa paroi antérieure [11]. Le traitement du papillome inversé associé à un carcinome prévalent doit associer un traitement chirurgical par voie externe, suivi d'une radiothérapie [12]. Sur les faibles effectifs des séries disponibles, la chimiothérapie néo-adjuvante n'augmente pas la survie.

Le pronostic est sévère et dépend de l'extension initiale [13]. Dans le cas où le carcinome est découvert à l'examen anatomopathologique définitif, l'attitude est discutée. Si le foyer de carcinome est de type invasif et limité à une zone anatomique sans foyers de dysplasie multiple par ailleurs, un complément de chirurgie large avec exérèse des parois osseuses de la zone en cause sera proposé et une irradiation complémentaire discutée. Dans le même cas, et si le carcinome est déclaré in situ, on réalise une surveillance rapprochée. Si le carcinome est étendu ou en foyers multiples ou encore associé à de nombreux foyers de dysplasie, cela signe une maladie dégénérative de la muqueuse, qui doit faire discuter une radiothérapie éventuellement combinée à une chimiothérapie, versus une chirurgie carcinologique large, ici à type d'exérèse ethmoido-frontale par voie mixte en collaboration avec l’équipe de neurochirurgie, suivie d'une irradiation complémentaire [11].

Les papillomes inversés se caractérisent par un risque élevé de récidive locale après chirurgie. Le diagnostic de ces récidives est difficile car bien souvent les patients sont asymptomatiques. C'est la surveillance, fondée sur le couple clinique-imagerie, qui permet d'en suspecter le diagnostic. Sur un plan thérapeutique, le choix de la voie d'abord se fait comme dans un cas de chirurgie de première intention en fonction de la topographie de la lésion et de ses extensions. Une récidive frontale ou maxillaire conduira cependant plus volontiers à une voie externe qu'en première intention. Les difficultés chirurgicales sont les mêmes que dans toutes les reprises chirurgicales au niveau des sinus et sont liées au manque de balises anatomiques et aux remaniements osseux et fibreux [10].

## Conclusion

Bien que tumeur rare des cavités naso-sinusiennes, le papillome inversé mérite les nombreuses publications qui lui ont été consacrées. En effet, il possède trois caractéristiques qui lui sont particulières: la destruction osseuse, le taux élevé de récidives post-opératoires et l'association synchrone ou métachrone avec un carcinome épidermoïde. L'imagerie intervient, non pas dans le diagnostic positif du papillome inversé, qui est histologique, apporté par la biopsie endonasale, mais dans l’établissement d'un diagnostic d'extension précis, ce qui déterminera la technique opératoire la plus adéquate: exérèse endoscopique ou chirurgie externe ou l'association des deux modalités. Les récidives constituent le principal échec de la chirurgie du papillome inversé; cependant, il n'existe pas de facteurs prédictifs de récidive. La surveillance reposant sur l'endoscopie et l'imagerie doit être rapprochée, notamment pendant les deux premières années post-opératoires.
